# Transcription factors Asg1p and Hal9p regulate pH homeostasis in *Candida glabrata*

**DOI:** 10.3389/fmicb.2015.00843

**Published:** 2015-08-18

**Authors:** Jing Wu, Xiulai Chen, Lijun Cai, Lei Tang, Liming Liu

**Affiliations:** ^1^State Key Laboratory of Food Science and Technology, Jiangnan UniversityWuxi, China; ^2^The Key Laboratory of Industrial Biotechnology, Ministry of Education, Jiangnan UniversityWuxi, China

**Keywords:** *Candida glabrata*, transcription factors, Asg1p, Hal9p, acid tolerance

## Abstract

*Candida glabrata* is an important microorganism used in commercial fermentation to produce pyruvate, but very little is known about its mechanisms for surviving acid stress in culture. In this study, it was shown that transcription factors Asg1p and Hal9p play essential roles in *C. glabrata* in the tolerance of acid stress, as the deletion of *CgASG1* or *CgHAL9* resulted in the inability to survive in an acidic environment. *Cgasg1*Δ and *Cghal9*Δ mutant strains are unable to maintain pH homeostasis, as evidenced by a decrease in intracellular pH and an increase in reactive oxygen species production, which results in metabolic disorders. The results showed that intracellular acidification was partly due to the diminished activity of the plasma membrane proton pump, CgPma1p. In addition, transcriptome sequencing revealed that *Cgasg1*Δ and *Cghal9*Δ mutant strains displayed a variety of changes in gene expression under acidic conditions, including genes in the MAPK signaling pathway, plasma membrane, or cell wall organization, trehalose accumulation, and the RIM101 signaling pathway. Lastly, quantitative reverse-transcribed PCR and cellular localization showed that CgAsg1p and CgHal9p played independent roles in response to acid stress.

## Introduction

*Candida glabrata*, a kind of haploid, asexual, ascomycetous yeast, is a major industrial microorganism that is used to produce organic acids, such as fumaric acid (Chen et al., 2015), malic acid (Chen et al., 2013), and α-ketoglutaric acid (Huang et al., 2006; Liang et al., 2008). Furthermore, *C. glabrata* is also the only microorganism used in commercial fermentation to produce pyruvate, which is widely used as a nutraceutical in the pharmaceutical and agrochemical fields and, recently, as the key metabolic precursor to the second-generation biofuels isobutanol and 3-methyl-1-butanol (Stanko et al., 1994; Atsumi et al., 2008). During pyruvate production, the pH of *C. glabrata* cultures gradually decreases due to acid accumulation. As a result, cell growth and acid production slow or even halt (Schügerl, 2000). The traditional method of solving this problem is to maintain the ambient pH by adding alkaline materials, such as NaOH, CaCO_3_, and Na_2_CO_3_, to the culture broth. However, this does not solve the problem fundamentally. Compared with *Saccharomyces cerevisiae*, the mechanism of tolerance to acid stress has not been investigated extensively in *C. glabrata*. Proteomic analysis of the pH response revealed that *C. glabrata* perceives low pH as less stressful than high pH (Schmidt et al., 2008). Studies on GPI-linked aspartyl proteases showed that CgYps1 is required to survive in low external pH environments by regulating the activity of the plasma membrane proton pump, CgPma1 (Bairwa and Kaur, 2011; Bairwa et al., 2014). Here, *S. cerevisiae* transcription factor Asg1p and Hal9p orthologs were functionally characterized from 41 zinc cluster proteins in *C. glabrata* to elucidate the pH-regulating mechanism more clearly (Klimova et al., 2014).

Deletion of either *S. cerevisiae ASG1* (*ScASG1*, YIL130W) or *C. albicans ASG1* (*CaASG1*, CaO19.166, CaO19.7800) resulted in reduced ability of growing on non-fermentable carbon sources (Kumar et al., 2000; Akache et al., 2001; Coste et al., 2008). *C. glabrata ASG1* (*CgASG1*, CAGL0G08844g), deletion of which led to an increased tolerance to salt stress (Klimova et al., 2014), has 50% sequence identity to *ScASG1* and 39% sequence similarity to *CaASG1*. Unexpectedly, CgAsg1p eliminated the conservative function of sustaining growth on non-fermentative carbon sources, but developed the ability to maintain growth under acid-stress conditions. Additionally, deletion of *S. cerevisiae HAL9* (*ScHAL9*, YOL089C) may lead to a decline in sodium and lithium tolerance and *ENA1* (Na^+^/Li^+^ extrusion pump) gene expression (Mendizabal et al., 1998; Pearson and Schweizer, 2002; Contador et al., 2011; Krauke and Sychrova, 2011). Although transcription factors from different fungal species respond similarly to diverse environmental conditions (Gasch, 2007), they still display species-specific functions because of their different environmental niches and several 100 million years of phylogenetic distance. Deletion of *C. glabrata HAL9* (*CgHAL9*, CAGL0I07755g), which has 43% sequence identity to *ScHAL9*, not only maintained the ability to grow on hyperosmotic medium, but also led to a new function that enabled growth under acidic conditions.

In this study, the functions of CgAsg1p and CgHal9p were identified in the regulation of pH homeostasis. To achieve this, different parameters of the mutant strains *Cgasg1*Δ and *Cghal9*Δ were analyzed with respect to those of *C. glabrata* ATCC 2001 (wild-type strain) under acidic conditions. Cell viability was decreased by diminishing plasma-membrane proton pump (H^+^-ATPase) activity, which influenced the intracellular pH (pH_in_) and reactive oxygen species (ROS). In addition, green fluorescent protein (GFP) fusion proteins and RNA-sequencing (RNAseq) were used to gain further insights into pH signaling and homeostasis pathways. Furthermore, the relationship between CgAsg1p and CgHal9p was studied by examining their expression and protein localization in wild-type, *Cgasg1*Δ, and *Cghal9*Δ strains.

## Materials and methods

### Strains, medium, and growth conditions

All strains and plasmids used in this study are listed in Table 1. YNB (0.67% yeast nitrogen base, 2% glucose, pH 5.2) medium was used to incubate *C. glabrata* strains in all experiments, except the utilization test of non-fermentable carbon sources in the *Cgasg1*Δ strain. YPD (1% yeast nitrogen base, 2% tryptone, 2% glucose) medium was used to determine cell viability. Strains *C. glabrata* ATCC 2001 (wild-type strain, *wt*) and *C. glabrata* ATCC 55 were gifts from Karl Kuchler. All *C. glabrata* strains were incubated at 30°C.

**Table 1:** **Strains and plasmids used in this study**.

**Strains or plasmids**	**Relevant characteristics**	**References**
**STRAINS**		
*C. glabrata* ATCC 2001	Wild-type strain	Roetzer et al., 2008
*C. glabrata* ATCC 55	*his3*Δ*trp1*Δ*ura3*Δ mutant strain	Roetzer et al., 2008
*Cgasg1*Δ	*C. glabrata* ATCC 55 (*asg1*Δ*::CgURA3*)	This study
*Cgasg1*Δ/*CgASG1*	*Cgasg1*Δ (pY13-*CgASG1*)	This study
*Cgasg1*Δ/*CgASG1-GFP*	*Cgasg1*Δ (pY13-*CgASG1*-*GFP*)	This study
*Cghal9*Δ	*C. glabrata* ATCC 55 (*hal9*Δ*::CgURA3*)	This study
*Cghal9*Δ/*CgHAL9*	*Cghal9*Δ (pY13-*CgHAL9*)	This study
*Cghal9*Δ/*CgHAL9-GFP*	*Cghal9*Δ (pY13-*CgHAL9*-*GFP*)	This study
*C. glabrata* ATCC 55/*GFP*	*C. glabrata* ATCC 55 (pY13-*GFP*)	This study
*Cgasg1*Δ*hal9*Δ	*Cgasg1*Δ (*hal9*Δ*::CgHIS3*)	This study
**PLASMIDS**		
pYES2	2μ, *Amp, URA3*, P_GAL_	Invitrogen (California, USA)
pY13	CEN6/ARSH4, *Amp, HIS3*, P_TEF_	Turbo (Beijing, China)
pY13-*CgASG1*	CEN6/ARSH4, *Amp, HIS3*, P_TEF_-*CgASG1*	This study
pY13-*CgASG1*-*GFP*	CEN6/ARSH4, *Amp, HIS3*, P_TEF_-*CgASG1*-*GFP*	This study
pY13-*CgHAL9*	CEN6/ARSH4, *Amp, HIS3*, P_TEF_-*CgHAL9*	This study
pY13-*CgHAL9*-*GFP*	CEN6/ARSH4, *Amp, HIS3*, P_TEF_-*CgHAL9*-*GFP*	This study
pY13-*GFP*	CEN6/ARSH4, *Amp, HIS3*, HIS3, P_TEF_-*GFP*	This study

The mutant strain *Cgasg1*Δ was obtained by genomic integration (Baudin et al., 1993). PCR products of the marker gene *CgURA3* and the 5′ and 3′ regions flanking of *CgASG1* were amplified from the genome of *wt* and the flanking PCR product was generated by fusion PCR. After transformed into the strain *C. glabrata* ATCC 55, the fusion fragment was integrated into the genome and the correct homologous recombination was verified by genomic PCR and DNA sequencing. The mutant strain *Cgasg1*Δ was constructed in the same way as *Cgasg1*Δ. *Cgasg1*Δ*hal9*Δ was obtained with the marker gene *CgHIS3* in the background of *Cgasg1*Δ.

Gene *CgASG1* and *CgHAL9* were amplified from the genome of *wt* and *GFP* fragment was amplified from the plasmid pYES2. The fragments *CgASG1*-*GFP* and *CgHAL9*-*GFP* were constructed by fusion PCR. Gene *CgASG1, CgHAL9, CgASG1*-*GFP*, and *CgHAL9*-*GFP* were expressed under the control of TEF1 promoter in *Cgasg1*Δ and *Cghal9*Δ to construct strains *Cgasg1*Δ/*CgASG1, Cghal9*Δ/*CgHAL9, Cgasg1*Δ/*CgASG1*-*GFP, Cghal9*Δ/*CgHAL9*-*GFP, Cgasg1*Δ/*CgHAL9*-*GFP*, and *Cghal9*Δ/*CgASG1*-*GFP*.

### Tolerance assays

The growth of *C. glabrata* strains under different stress conditions was assayed qualitatively by spotting 4 μL of tenfold dilutions of logarithmic-phase yeast broth cultures onto YNB plates containing different carbon sources and different concentrations of LiCl and NaCl, or YNB plates at different pH, as described previously (Sanglard et al., 1999). After incubation at 30°C for 4 days, colonies were easily visualized on the plates.

### Growth analysis and viability measurement

For growth analysis, logarithmic-phase *C. glabrata* cells were inoculated at an initial OD_600_ of 0.1 in YNB medium adjusted to pH 2.0–9.0. The absorbance of the cultures was recorded at 600 nm at regular time intervals, and the growth curve was plotted as the OD_600_ over time. For viability measurement, appropriate dilutions of *C. glabrata* cells in YNB and YNB-pH 2.0 media were plated onto YPD plates at various time points, and total colony-forming units (cfus) were calculated by counting colonies that appeared after a 2-day incubation at 30°C. A histogram was made to illustrate the survival percentage over time.

### pHluorin calibration and intracellular pH measurement

In *S. cerevisiae*, the pHluorin calibration and intracellular pH were measured as described previously (Bracey et al., 1998). The same protocol was optimized for *C. glabrata* with the fluorescent probe 5(6)-carboxyfluorescein diacetate succinimidyl ester (CFDA-SE, Sigma-Aldrich, St. Louis, MO, USA) (Bairwa and Kaur, 2011). After incubated in YNB or YNB-pH 2.0 medium for 2 h, log-phase *C. glabrata* cells were collected, washed and resuspended in 50 mM citric/phosphate (CP) buffer (pH 4.0) to an OD_600_ of 0.5. Add CFDA-SE to a final concentration of 150 μM and incubate the cell suspension at 37°C for 1 h to load the probe. After removed the unloaded probe with CP buffer, the fluorescence intensity was measured by a spectrofluorophotometer (Shimadzu RF-5310PC, Tokyo, Japan) with excitation at 430 and 490 nm and emission at 525 nm. The intracellular pH could be calculated with the fluorescence intensity by a calibration curve. Log-phase wild-type cells were incubated in 50 mM CP buffer at pH 4.0–7.5 (0.5 units per interval) with CFDA-SE to load the probe and 0.5 mM carbonyl cyanide m-chlorophenyl hydrazone (CCCP; Sigma-Aldrich) to make the intracellular pH similar to the extracellular pH. Fluorescence intensities were measured, and a calibration curve of the ratio of the intensities at 490 and 430 nm vs. pH was plotted.

### Intracellular ROS measurement

ROS production was measured using the non-fluorescent probe 2′, 7-dichlorodihydrofluorescein diacetate (DCFH-DA, Sigma-Aldrich) (Bairwa and Kaur, 2011). After incubated in YNB or YNB-pH 2.0 medium for 2 h, log-phase *C. glabrata* cells were collected, washed, and diluted to an OD_600_ of 1.0 in phosphate-buffered saline (PBS). Add DCFH-DA to a final concentration of 100 μM and incubate the cell suspension at 28°C for 30 min to load the probe. DCFH-DA could convert into DCFH by cellular esterases, and then oxidized to the fluorescent compound 2′, 7-dichlorofluorescein (DCF) by intracellular H_2_O_2_. After washed and resuspended in 100 μL of PBS, and the fluorescence intensity of the cell suspension was measured using a fluorescence spectrometer (RF-5310PC, Shimadzu) with excitation at 480 nm and emission at 530 nm. The fluorescence intensity was read directly as arbitrary units (Machida et al., 1998). ROS production was expressed as the ratio relative to wild-type cells in YNB.

### Plasma membrane H^+^-ATPase activity assay

Plasma membrane suspensions were prepared, as described previously (Fernandes et al., 1998; Nakamura et al., 2001). After incubated in YNB or YNB-pH 2.0 medium for 2 h, log-phase *C. glabrata* cells were harvested and suspended in 1 mL solution (100 mM Tris, 5 mM EDTA, and 2 mM dithiothreitol) to an OD_600_ of 20. Protease inhibitor cocktail (Roche, Shanghai, China) was added to inhibit protease activity. Cells were lysed by ultrasonic disruptor and diluted with 5 mL solution (0.1 M Tris-HCl, 0.33 M sucrose, 5 mM EDTA, and 2 mM dithiothreitol). Centrifugate the solution (1000 g, 3 min, 4°C), collecte the supernatant and centrifuge (3000 g, 5 min, 4°C), followed by another centrifugation (19,000 g, 45 min, 4°C) to obtain the total membrane fraction. The pellet was resuspended in 100 μL solution (10 mM Tris-HCl, 20% glycerol, 0.1 mM EDTA and 0.1 mM dithiothreitol) and stored at −70°C. The protein concentration of the total membrane fraction was determined by the Bradford method.

Plasma membrane H^+^-ATPase activity was assayed as described previously (Viegas et al., 1995). The total membrane fraction (5 μg) was incubated at 30°C in 120 μL solution (5 mM ATP, 10 mM MgSO_4_, 50 mM KCl, and 50 mM MES). The mixture (50 mM KNO_3_, 5 mM NaN_3_, and 0.2 mM ammonium molybdate) was added to eliminate the activity of non-plasma membrane ATPases. After 30 min, 130 μL solution (1% SDS, 0.6 M H_2_SO_4_, 1.2% ammonium molybdate and 1.6% ascorbic acid) was added to stop the reaction. After 10 min, the amount of released inorganic phosphate (Pi) was measured at 750 nm. The ATPase activity of the plasma membrane was expressed in micromoles of Pi released per minute per milligram of total membrane protein.

### Protein localization

Fluorescence experiments were performed as described previously (Görner et al., 1998). CgAsg1p-GFP and CgHal9p-GFP fusion proteins were expressed under the control of the TEF1 promoter to examine the subcellular localization of CgAsg1p and CgHal9p. Log-phase cells were inoculated to an initial OD_600_ of 1.0 in either YNB or YNB-pH 2.0 media, and incubated at 30°C for 2 h. Samples were collected, and images were taken with a fluorescence microscope (Nikon Eclipse 80i, Tokyo, Japan), coupled with a Nikon DS-Ri1 digital camera.

### RNA-sequencing transcriptome analysis

Six different *C. glabrata* RNA libraries were prepared for sequencing. Wild-type and *Cgasg1*Δ and *Cghal9*Δ cells were grown in YNB medium to exponential phase, and exposed to YNB or YNB-pH 2.0 media at 30°C for 2 h. Total RNA was extracted using the TaKaRa MiniBEST Universal RNA Extraction Kit (TaKaRa) and stored quickly at −80°C. The quality of the RNA was checked with a K5500 micro-spectrophotometer and Agilent 2100 Bioanalyzer (Agilent Technologies, Santa Clara, CA, USA). These frozen samples were sent to Biomarker Technologies (Beijing, China) (http://www.biomarker.com.cn), which provides services of global gene analysis. The NEBNext Poly (A) mRNA Magnetic Isolation Module (New England Biolabs (NEB), Ipswich, MA, USA, E7490) was used to remove rRNA and enrich mRNA. The NEBNext mRNA Library Prep Master Mix Set for Illumina (NEB, E6110) and NEBNext Multiplex Oligos for Illumina (NEB, E7500) were used to construct the libraries. The qualified libraries generated clusters on an Illumina cbot (Illumina, San Diego, CA, USA), and sequencing was performed on an Illumina HiSeq™ 2500. The data discussed in this study are available at http://www.ncbi.nlm.nih.gov/sra/?term=SRP055793.

### qRT-PCR analysis

Log-phase *C. glabrata* cells were inoculated in YNB or YNB-pH 2.0 media to an initial OD_600_ of 2.0 and incubated for 2 h. Then, cells were harvested, and total RNA was extracted using the MiniBEST Universal RNA Extraction Kit (TaKaRa, Dalian, Liaoning, China) according to the manufacturer's instructions. cDNA was synthesized from total RNA using the PrimeScript™ II 1st Strand cDNA Synthesis Kit (TaKaRa), and the relative amount of specific mRNA was determined by qRT-PCR (Nolan et al., 2006). qRT-PCR was performed using an iQ5 Continuous Fluorescence Detector System (Bio-Rad, Hercules, CA, USA) using the SYBR® *Premix Ex Taq*™ (TaKaRa). All experiments were performed in triplicate, and the data were normalized using the actin gene as a control.

## Results

### *CgASG1* and *CgHAL9* are essential for survival in acid-stress conditions in *C. glabrata*

**Figure 1:**
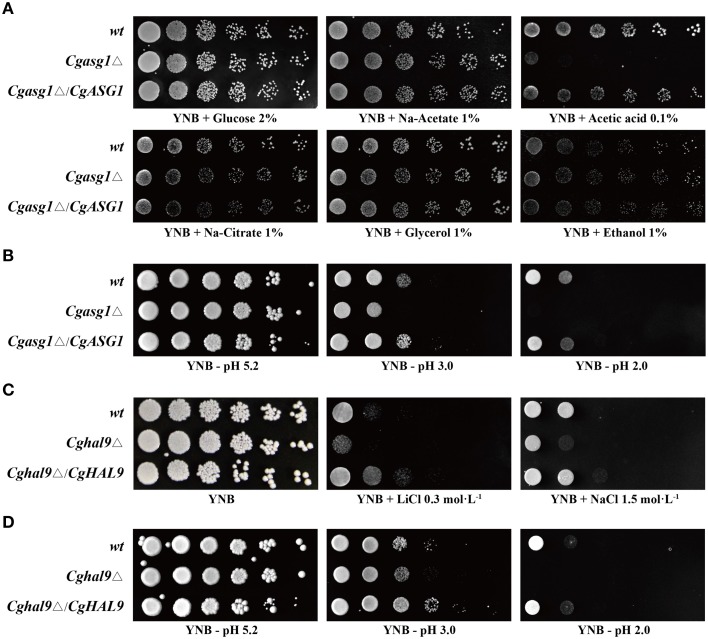
**Growth assays in different YNB media. (A)** Deletion of *CgASG1* has no effect on the utilization of non-fermentative carbon sources in *C. glabrata*. **(B)** CgAsg1p plays a role under acid-stress conditions. **(C)** *CgHAL9* serves an important role in cell growth under hypersaline conditions in *C. glabrata*. **(D)** CgHal9p plays a role under acid-stress conditions. Logarithmic-phase cells of each *C. glabrata* strain were adjusted to 2 × 10^7^ cells/mL, and then 4 μL of serial tenfold dilutions were spotted onto the corresponding YNB media, as indicated. Pictures were taken after 4 days of growth at 30°C.

A *Cgasg1*Δ mutant strain was constructed as a first step to test the function of CgAsg1p. The growth of wild-type and *Cgasg1*Δ strains was tested on solid media containing various carbon sources. The *Cgasg1*Δ strain exhibited the same phenotype as the wild-type strain on media containing sodium acetate, sodium citrate, glycerol, and ethanol, but a drastically defective phenotype was displayed on plates using acetic acid as a carbon source (Figure 1A). A reduction in pH contributed to the growth defect on acetic acid. Therefore, the growth phenotype was examined on YNB medium over a pH range from 2.0 to 9.0. The *Cgasg1*Δ strain exhibited the same growth as the wild-type strain from pH 4.0 to 9.0, but displayed growth inhibition from pH 2.0 to 3.0. The complemented strain *Cgasg1*Δ/*CgASG1* showed the same growth as the wild-type strain (Figure 1B and Figure S1A). These results demonstrated that CgAsg1p is involved in the acid-stress response.

The *Cghal9*Δ mutant strain was constructed to investigate whether *CgHAL9* is also involved in the osmotic stress response. The *Cghal9*Δ mutant strain exhibited a defective growth phenotype on YNB medium containing LiCl (0.3 mol·L^−1^) or NaCl (1.5 mol·L^−1^), while the complemented strain *Cghal9*Δ/*CgHAL9* showed the same growth as the wild-type strain, suggesting CgHal9p played a vital role in salt tolerance (Figure 1C). Because transcription factors from different fungal species can share similar structural domains, but still display species-specific functions, additional phenotypic assays were performed on YNB medium from pH 2.0 to 9.0. Coincidentally, the *Cghal9*Δ mutant strain displayed the same growth as the wild-type strain from pH 4.0 to 9.0, while it exhibited growth inhibition from pH 2.0 to 3.0. The *Cghal9*Δ/*CgHAL9* strain showed the same growth as the wild-type strain from pH 2.0 to 9.0 (Figure 1D and Figure S1B). These results indicated that CgHal9p also plays a role in pH tolerance.

### Deletion of *CgASG1* or *CgHAL9* decreases growth in a low-pH environment

**Figure 2:**
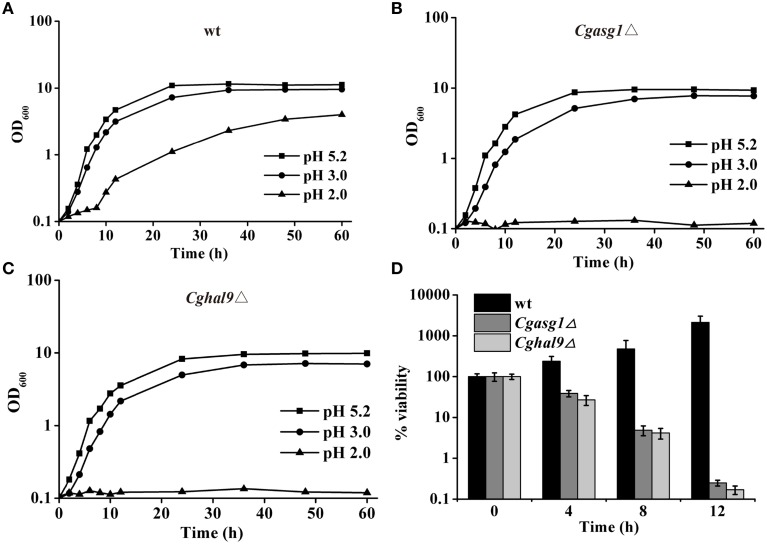
*****CgASG1*** and ***CgHAL9*** are required to survive the low external pH environment**. Growth curves of wild-type **(A)**, *Cgasg1*Δ **(B)**, and *Cghal9*Δ **(C)** strains were analyzed in YNB, YNB-pH 2.0, and YNB-pH 3.0 media. Data are shown as mean values of three independent experiments. **(D)** Time-kill analysis of the *Cgasg1*Δ and *Cghal9*Δ strains in an acidic environment. Data are expressed as the percentages of viability of each strain relative to that of the control (time point 0) in YNB medium. The means and standard deviations for three independent experiments are shown.

The growth of the wild-type, *Cgasg1*Δ, and *Cghal9*Δ strains were examined in YNB medium and YNB medium at pH 2.0 and pH 3.0, and were compared after 24 h. For the wild-type strain, growth in YNB medium at pH 2.0 and 3.0 was 90 and 34% slower, respectively, than in YNB medium (Figure 2A). Deletion of *CgASG1* or *CgHAL9* led to a 15 or 13% reduction of growth, respectively, in YNB medium (Figures 2A–C). For the *Cgasg1*Δ strain, growth was reduced by 41% at pH 3.0, while no growth was observed at pH 2.0 (Figure 2B). Similarly, for the *Cghal9*Δ strain, there was a 40% reduction in growth at pH 3.0, while no growth was observed at pH 2.0 (Figure 2C).

To further probe the reduction in the growth capacity of the *Cgasg1*Δ and *Cghal9*Δ mutant strains in an acidic environment, cell viability was measured after incubation in YNB and YNB-pH 2.0 media. The wild-type strain showed a constantly increasing viability during cultivation in YNB-pH 2.0 medium. However, both the *Cgasg1*Δ and *Cghal9*Δ strains exhibited a 90% reduction in cfus after incubation in YNB-pH 2.0 medium for 8 h, while the overexpression of the deleted genes rescued this growth defect, as expected (Figure 2D and Table S1). These data indicated that deletion of either *CgASG1* or *CgHAL9* was deleterious to cell viability under acidic conditions.

### Diminished H^+^-ATPase activity contributes to the reduced intracellular pH and increased ROS production under acidic conditions

**Figure 3:**
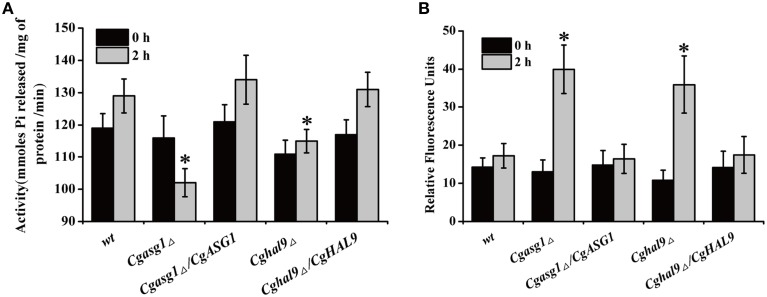
**Analysis of reduced cell viability by measurement of CgPma1 activity and intracellular ROS content. (A)** H^+^-ATPase activity assay of *Cgasg1*Δ and *Cghal9*Δ strains in YNB and YNB-pH 2.0 media. H^+^-ATPase activity was expressed as micromoles of Pi released per minute per milligram of total membrane protein. **(B)** After incubation for 2 h in YNB or YNB-pH 2.0 media, the intracellular ROS content of wild-type, *Cgasg1*▵, and *Cghal9*▵ strains were measured as described in the Materials and Methods Section. The above data are shown as the defined units at 0 and 2-h time points for incubation in YNB and YNB-pH 2.0 media. The means and standard deviations for three independent experiments are shown. Error bars represent standard deviations. (^*^P < 0.05 compared to the corresponding wild-type control, as determined by *t*-test).

To explain the aforementioned decrease in cell viability, the plasma-membrane proton pump (H^+^-ATPase) activity of the wild-type, *Cgasg1*Δ, and *Cghal9*Δ strains were measured after treatment for 2 h in YNB and YNB-pH 2.0 media. Incubating the wild-type strain in YNB-pH 2.0 medium led to an 8% rise in H^+^-ATPase activity (Figure 3A). Unexpectedly, *Cgasg1*Δ strain exhibited a 10% reduction in H^+^-ATPase activity in YNB-pH 2.0 medium while *Cghal9*Δ exhibited the same change in H^+^-ATPase activity as the wild-type strain. Furthermore, the complemented strains *Cgasg1*Δ/*CgASG1* and *Cghal9*Δ/*CgHAL9* displayed the same H^+^-ATPase activities as the wild-type strain in YNB and YNB-pH 2.0 media (Figure 3A). The results led us to consider how the decreased H^+^-ATPase activity influenced cell viability.

Because intracellular ROS production is closely related to intracellular pH, which could be reduced by decreased H^+^-ATPase activity, the intracellular pH and ROS content of logarithmically growing wild-type, *Cgasg1*Δ and *Cghal9*Δ strains were measured after incubation for 2 h in YNB and YNB-pH 2.0 media. In the wild-type strain, a pH_in_ of approximately 6.0–6.2 (Table 2) and a consistent intracellular ROS content were maintained in YNB and YNB-pH 2.0 media (Figure 3B). The *Cgasg1*Δ and *Cghal9*Δ mutant strains maintained a pH_in_ of 6.0 in YNB medium (Table 2); however, in YNB-pH 2.0 medium, the pH_in_ decreased to 5.27 and 5.31, respectively (Table 2). The *Cgasg1*Δ and *Cghal9*Δ strains showed the same ROS levels as the wild-type strain in YNB medium, but exhibited twofold higher ROS levels in YNB-pH 2.0 medium (Figure 3B). Notably, the complemented strains *Cgasg1*Δ/*CgASG1* and *Cghal9*Δ/*CgHAL9* showed the same pH_in_ levels and ROS levels as the wild-type strain in YNB and YNB-pH 2.0 media (Table 2 and Figure 3B). The above results suggest that the deletion of either *CgASG1* or *CgHAL9* led to the inhibition of cell viability, which was induced by a reduction in intracellular pH following an increase in ROS production in YNB-pH 2.0 medium.

**Table 2:** **Intracellular pH of wild-type, mutant and complemented strains of *C. glabrata* as below^a^**.

***C.glabrata* strain**	***wt***	***Cgasg1*Δ**	***Cgasg1*Δ/*CgASG1***	***Cghal9*Δ**	***Cghal9*Δ/*CgHAL9***
YNB	6.13 ± 0.17	5.98 ± 0.21	6.08 ± 0.16	6.05 ± 0.24	6.10 ± 0.19
YNB-pH2.0	6.09 ± 0.20	5.27 ± 0.23	6.16 ± 0.21	5.31 ± 0.15	6.12 ± 0.13

a *Data are represented as mean values of three independent experiments*.

### Nuclear localization of CgAsg1p and CgHal9p is stress regulated

**Figure 4:**
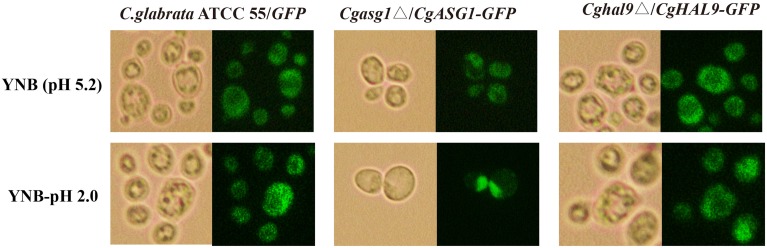
**CgAsg1p, but not CgHal9p, accumulates in the nucleus under acid-stressed conditions**. *CgASG1* and *CgHAL9* were fused to the green fluorescent protein (GFP) and expressed in the *Cgasg1*Δ and *Cghal9*Δ strains, respectively, under the control of the TEF1 promoter. Log-phase cells were treated for 2 h in YNB or YNB-pH 2.0 media, and the localization of the GFP fusions was determined by fluorescence microscopy.

*Cgasg1*Δ/*CgASG1-GFP* and *Cghal9*Δ/*CgHAL9*-*GFP* strains were constructed, and the subcellular localization of CgAsg1p and CgHal9p in YNB and YNB-pH 2.0 media were observed by fluorescence microscopy. In the control strain *C. glabrata* ATCC 55/*GFP*, GFP fluorescence was observed to be cytoplasmic in both YNB and YNB-pH 2.0 media (Figure 4). In the *Cgasg1*Δ strain, the fluorescence signal was localized in the cytoplasm in YNB medium, but in YNB-pH 2.0 medium, the fluorescence was gradually transferred from the cytoplasm to the nucleus (Figure 4). In the *Cghal9*Δ strain, the GFP fusion protein was constitutively enriched in the cytoplasm in YNB and YNB-pH2.0 media. These results suggest that CgAsg1p and CgHal9p play roles in different ways in resistance to acid stress.

### Gene transcription profiling in response to a low environmental pH

RNAseq was performed on logarithmically growing wild-type, *Cgasg1*Δ, and *Cghal9*Δ strains after incubation for 2 h in YNB or YNB-pH 2.0 media. The complete dataset can be found at Sequence Read Archive http://www.ncbi.nlm.nih.gov/sra/?term=SRP055793. Differentially regulated genes (≥ two-fold change with an FDR < 0.01) were analyzed for co-regulation by hierarchical clustering and annotated with the gene ontology (GO) term for biological processes.

**Figure 5:**
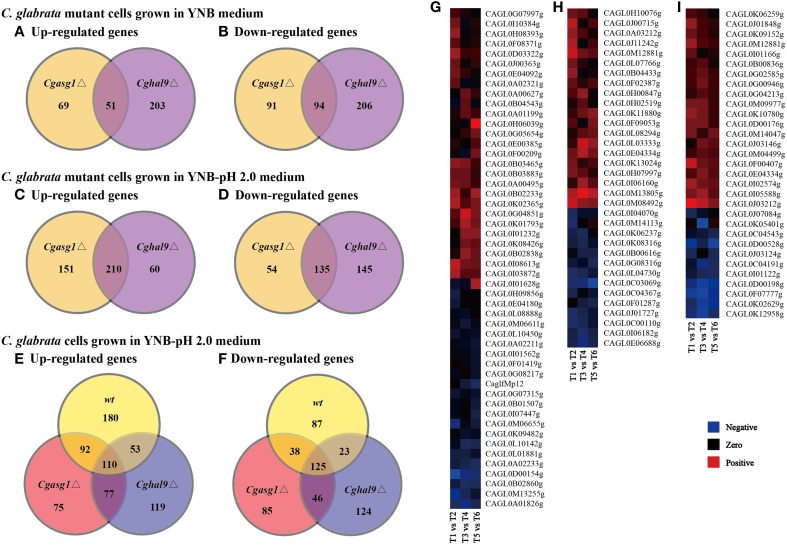
**Comparison of genome-wide expression levels in response to acid stress**. Venn diagram depicting the overlap between up-regulated **(A)** and down-regulated **(B)** genes in the *Cgasg1*Δ and *Cghal9*Δ strains, compared with the wild-type strain in YNB medium. Venn diagram depicting the overlap between up-regulated **(C)** and down-regulated **(D)** genes in the *Cgasg1*Δ and *Cghal9*Δ strains compared with the wild-type strain in YNB-pH 2.0 medium. Venn diagram depicting the overlap between up-regulated **(E)** and down-regulated **(F)** genes in wild-type (T2), *Cgasg1*Δ (T4), and *Cghal9*Δ (T6) in YNB-pH 2.0 medium compared with the corresponding strains (T1, T3, T5) in YNB medium. Comparison of gene expression involved in cellular component of integral component of membrane (GO:0016021) **(G)**, cellular component of membrane (GO:0016020) **(H)**, and oxidation-reduction process (GO:0055114) **(I)** in T2, T4, and T6 compared with T1, T3, and T5, respectively.

First, comparing the transcript profiles of the *Cgasg1*Δ and *Cghal9*Δ mutant strains to that of the wild-type strain in YNB medium, a total of 305 and 554 genes were found to be differentially regulated, respectively (Figures 5A,B). Among this gene set, a striking overlap of 51 induced and 94 repressed genes were common to both the *Cgasg1*Δ and *Cghal9*Δ strains (Figures 5A,B). The common induced gene set included genes belonging to tricarboxylic acid cycle (GO:0006099), transmembrane transport (GO:0055085), integral component of membrane (GO:0016021) and component of fungal-type cell wall (GO:0009277) (Supplementary Data Sheet 1). Genes associated with glycolysis (GO:0006096) were significantly up-regulated only in the *Cgasg1*Δ strain, and genes involved in cell cycle (GO:0007049) were up-regulated only in the *Cghal9*Δ strain (Supplementary Data Sheet 1). The common repressed gene set included genes involved in translation (GO:0006412), structural constituent of ribosome (GO:0003735), protein transport (GO:0015031), proteolysis (GO:0006508), and ATP catabolic process (GO:0006200) (Supplementary Data Sheet 2). Additionally, genes implicated in adhesion to host (GO:0044406) were uniquely represented in the repressed gene dataset of the *Cghal9*Δ strain. These data suggest that CgAsg1p and CgHal9p exert a similar regulatory effect on biological processes.

Next, the transcript profiles of the *Cgasg1*Δ and *Cghal9*Δ mutant strains were also compared with that of the wild-type strain in YNB-pH 2.0 medium, and a strikingly overlapping set of 210 induced and 135 repressed genes were common to both the *Cgasg1*Δ and *Cghal9*Δ strains (Figures 5C,D). Furthermore, up-regulation of genes involved in chromatin modification (GO:0016568), DNA repair (GO:0006281) and mitosis (GO:0007067), as well as the down-regulation of genes implicated in response to stress (GO:0006950), rRNA processing (GO:0006364), and ribosome biogenesis (GO:0042254), was observed in the *Cgasg1*Δ and *Cghal9*Δ strains in response to acid stress (Supplementary Data Sheets 3, 4). Up-regulated genes involved in protein folding (GO:0006457) and autophagy (GO:0006914), as well as down-regulated genes involved in carbohydrate metabolic process (GO:0005975) and pyridoxal phosphate biosynthetic process (GO:0042823), were uniquely exhibited in the *Cgasg1*Δ strain. Up-regulated genes implicated in phospholipid metabolic process (GO:0006644), as well as down-regulated genes implicated in RNA metabolic process (GO:0016070) and glucose metabolic process (GO:0006006), were uniquely observed in the *Cghal9*Δ strain.

In addition, further GO enrichment analysis was conducted to compare the transcript profiles of the wild-type, *Cgasg1*Δ and *Cghal9*Δ strains grown in YNB-pH 2.0 medium with that of the corresponding strains grown in YNB medium. A total of 708, 648, and 677 genes were found to be differentially expressed, respectively. Among this gene set, 435, 354, and 359 genes were induced and 273, 294, and 318 genes were repressed in the wild-type, *Cgasg1*Δ, and *Cghal9*Δ strains, respectively (Figures 5E,F). In the wild-type strain, the up-regulated genes were involved in gluconeogenesis (GO:0006094), nucleic acid binding (GO:0003676), and trehalose catabolic process (GO:0005993), and the repressed genes were implicated in cell division (GO:0051301), chromosome segregation (GO:0007059) and translational termination (GO:0006415) (Supplementary Data Sheets 5, 6). Similar to the wild-type strain, the *Cgasg1*Δ and *Cghal9*Δ strains grown in YNB-pH 2.0 medium showed up-regulation of genes involved in oxygen transport (GO:0015671), nucleotide-excision repair (GO:0006289), and cellular component of nucleus (GO:0005634), and down-regulation of genes involved in oxidation-reduction process (GO:0055114), intracellular protein transport (GO:0006886), transmembrane transport (GO:0055085), and cellular component of fungal-type cell wall (GO:0009277), plasma membrane (GO:0005886) and vacuolar membrane (GO:0005774). In contrast to the wild-type strain, several up-regulated genes in the *Cgasg1*Δ and *Cghal9*Δ strains grown in YNB-pH 2.0 medium were implicated in biosynthetic process of dTTP (GO:0006235), dTMP (GO:0006231) and dUMP (GO:0006226) and catabolic process of dUTP (GO:0046081) and dITP (GO:0035863), and down-regulated genes implicated in trehalose biosynthetic process (GO:0005992), ATP catabolic process (GO:0006200), protein folding (GO:0006457) and protein metabolic process (GO:0019538). A lot of genes involved in cellular component of integral component of membrane (GO:0016021), cellular component of membrane (GO:0016020), and oxidation-reduction process (GO:0055114) were differently expressed in *wt, Cgasg1*Δ and *Cghal9*Δ mutant strains in response to acid stress (Figures 5G–I), which was in accordance with change in H^+^-ATPase activity, intracellular pH and ROS content. The RNAseq data indicated that the transcriptional responses of the *Cgasg1*Δ and *Cghal9*Δ mutant strains to acid stress partly differed from that of the wild-type strain.

**Figure 6:**
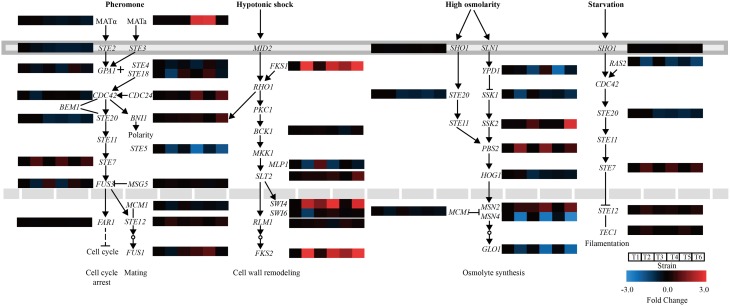
**Changes in gene expression and MAPK signaling pathways in ***Cgasg1***Δ and ***Cghal9***Δ strains**. Transcriptional profiling of genes involved in MAPK signaling pathways were compared, including the mating pheromone response, the cell wall integrity pathway, the spore wall assembly, the growth control pathway, and the HOG pathway. T1-T6 represent gene transcription levels of the wild-type, *Cgasg1*Δ and *Cghal9*Δ strains in YNB and YNB-pH 2.0 media, respectively.

Genes involved in MAPK pathways associated with the stress response were further analyzed. In the mating pheromone response, the lack of *CgASG1* or *CgHAL9* induced the overexpression of many genes, such as the upstream genes (*GPA1, STE18*, and *CDC24*) of the pheromone pathway, the final kinase gene *FUS3*, and the regulation of termination of mating projection growth gene *FUS1* (Figure 6). However, the expression of *STE20*, which also functions in the high osmolarity glycerol (HOG) pathway, decreased (Figure 6). In the HOG pathway, there was decreased expression of *YPD1*, a part of the branched *SLN1*-*YPD1*-*SKN7*/*SSK1* two-component regulatory system that controls the activity of the HOG1 pathway (Figure 6). Another gene, *HOG1*, which controls the transcriptional regulation of target genes via the stress response element (STRE), was also down-regulated in the *Cgasg1*Δ and *Cghal9*Δ mutant strains (Figure 6). These genome-scale characterizations revealed that changes in MAPK signaling pathways could partly explain the sensitivity to acid stress of the *Cgasg1*Δ and *Cghal9*Δ strains.

### Expression of acid stress-related genes

**Figure 7:**
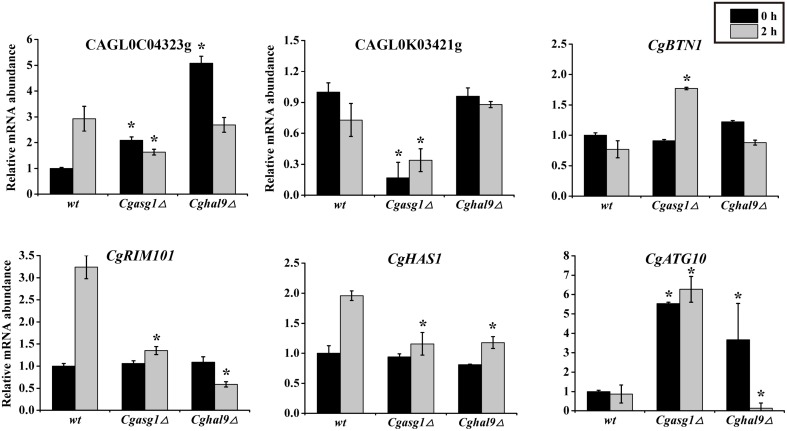
**Quantitative reverse-transcribed PCR (qRT-PCR) validation of transcriptional profiles in an acidic environment**. Logarithmic-phase *C. glabrata* cells were incubated in YNB or YNB-pH 2.0 media for 2 h. qRT-PCR analyses of the indicated genes was performed as described in the Materials and Methods Section. The means and standard deviations for three independent experiments are shown. *C. glabrata* strains: wild-type, *Cgasg1*Δ and *Cghal9*Δ strains. (^*^P < 0.05 compared to the corresponding wild-type control, as determined by *t*-test).

To verify the accuracy of the RNAseq data, a subset of gene responses to acid stress were further evaluated by qualitative reverse-transcribed PCR (qRT-PCR), including highly up- and down-regulated genes in the wild-type, *Cgasg1*Δ and *Cghal9*Δ strains. As shown in Figure 7, the reduction of CAGL0C04323g and CAGL0M10439g mRNA abundance indicated that the *Cgasg1*Δ and *Cghal9*Δ strains could not synthesize enough trehalose to cope with stress. In addition, the expression of *CgBTN1* (CAGL0J05104g), which is involved in vacuole pH homeostasis, was increased, while the expression of another gene, *CgRIM101* (CAGL0E03762g), which encodes a pH-response transcription factor, decreased in the two mutant strains, thereby further influencing the transcription of more acid-expressed genes in YNB-pH 2.0 medium. Additionally, the expression of ATP-dependent RNA helicase encoded gene, *CgHAS1* (CAGL0M13519g), which is involved in ribosome biogenesis, and a ubiquitin-like-conjugating enzyme encoded gene, *CgATG10* (CAGL0M13519g), which is involved in protein transport, were also altered in both mutant strains. This collectively suggests that CgAsg1p and CgHal9p play vital roles in the response to acid stress through various pathways, including trehalose synthesis, vacuole pH homeostasis, the RIM101 pH-response pathway, etc. Besides, other 6 genes were randomly selected and the mRNA level were measured by qRT-PCR. There was good agreement (*R* = 0.86) between the RNAseq data and the qRT-PCR results (Table S2 and Figure S2).

### The *Cgasg1*Δ*hal9*Δ double deletion strain exhibits a wild-type phenotype in YNB-pH 2.0 medium

**Figure 8:**
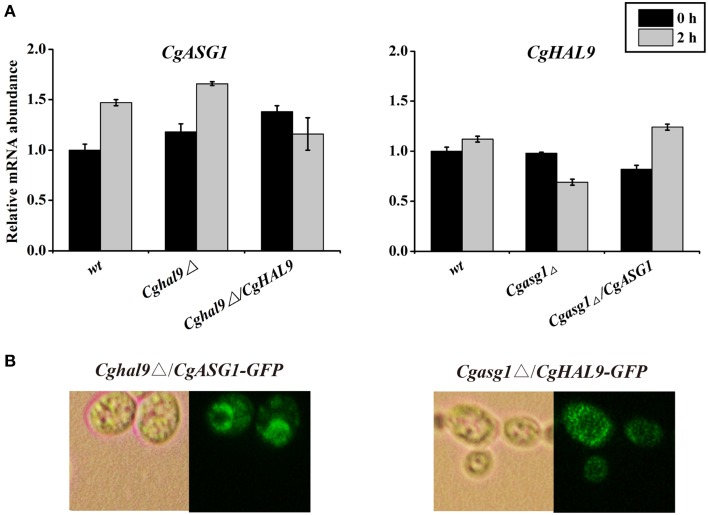
**An interaction between ***CgASG1*** and ***CgHAL9*** was tested using quantitative reverse-transcribed PCR (qRT-PCR) and green fluorescent protein (GFP) localization. (A)** qRT-PCR was performed on RNA samples of log-phase cells in YNB and YNB-pH 2.0 media to analyze the expression levels of *CgASG1* and *CgHAL9* in the indicated strains. Results are presented as fold expression relative to the levels of the wild-type β-actin control at 0 and 2-h time points of incubation in YNB and YNB-pH 2.0 media. The means and standard deviations for three independent experiments are shown. **(B)** Log-phase *Cgasg1*Δ/*CgHAL9*-*GFP* and *Cghal9*Δ/*CgASG1*-*GFP* cells were grown in YNB-pH 2.0 medium for 2 h, and the localization of GFP fusion proteins was determined by fluorescence microscopy.

First, qRT-PCR was performed to verify the above RNAseq results regarding *CgASG1* expression in the *Cghal9*Δ strain and *CgHAL9* expression in the *Cgasg1*Δ strain. The results showed that the expression of either gene was not affected by the lack of the other gene in YNB or YNB-pH 2.0 medium (Figure 8A), which naturally followed the deduction that *CgASG1* and *CgHAL9* might function in parallel to cope with acid stress. Next, *Cgasg1*Δ/*CgHAL9*-*GFP*, and *Cghal9*Δ/*CgASG1*-*GFP* strains were constructed, and the localization of CgAsg1p in the *Cghal9*Δ strain and the localization of CgHal9p in the *Cgasg1*Δ strain were detected. CgAsg1p-GFP was observed within the nucleus of the *Cghal9*Δ strain, while CgHal9p-GFP was observed in the cytoplasm of the *Cgasg1*Δ strain, showing that the localization of either transcription factor was not influenced by the other (Figure 8B). This further suggested that the two genes act independently of each other under both normal and acidic conditions.

Because the aforementioned results led to the conclusion that *CgASG1* and *CgHAL9* function independently to cope with a low-pH environment, we wondered whether cells were more sensitive to acid when both of the genes were deleted. To test this, *CgHAL9* was deleted in the *Cgasg1*Δ background, and the growth of the single and double mutants were monitored in YNB-pH 2.0 medium. Unexpectedly, the *Cgasg1*Δ*hal9*Δ double-deletion strain exhibited a wild-type growth phenotype in YNB-pH2.0 medium, despite the fact that each single mutant showed growth defects (Figure S3). We speculate that double deletion of *CgASG1* and *CgHAL9* might trigger a more effective way to respond to acid stress. The mechanism of this phenomenon is not fully understood and further research is needed to address this issue.

## Discussion

As the only industrial microorganism in the commercial, fermentative production of pyruvate (Yonehara and Miyata, 1994; Li et al., 2000; Liu et al., 2004), *C. glabrata* has not been exclusively investigated in the transcriptional regulatory response to acid-stress. Previous studies revealed that the transcription factors Msn2p and Msn4p are essential for resistance to various stresses in *C. glabrata* (Roetzer et al., 2008). Other studies on *C. glabrata* indicated that the transcription factors Yap1p, Skn7p, and Slm7p play vital roles in the resistance to oxidative stress (Cuéllar-Cruz et al., 2008; Briones-Martin-Del-Campo et al., 2014). Because studies in this field are so limited, our investigation of the transcription factors CgAsg1p and CgHal9p appears to be essential for elucidating the pH-regulating mechanism. Furthermore, the *S. cerevisiae* or *C. albicans* homologs of CgAsg1p and CgHal9p have not been previously reported to play a role in pH homeostasis.

This study focused on the functional identity of the novel roles played by CgAsg1p and CgHal9p in regulating pH homeostasis under acidic conditions. The tolerance of the *Cgasg1*Δ and *Cghal9*Δ strains to acid stress was apparent, as evidenced by decreases in H^+^-ATPase activity and intracellular pH, and increase in ROS production. It was found that the wild-type strain could maintain intracellular pH homeostasis in an acidic environment by increasing H^+^-ATPase activity, which was consistent with the notion that the fungal plasma membrane ATPase is tightly coupled for proton transportation and pH susceptibility (Serrano et al., 1986). However, the *Cgasg1*Δ and *Cghal9*Δ strains lost this ability, thus, the intracellular pH was reduced, resulting in an elevated ROS content, which is concordant with the finding that a reduced pH_in_ was accompanied by increased ROS production (Giannattasio et al., 2005). The cell viability of the *Cgasg1*Δ and *Cghal9*Δ strains was reduced, and this result was supported by the findings that ROS prevail over cellular defense systems and participate in the regulation of apoptosis through various components of the apoptotic machinery (Madeo et al., 1999; Landolfo et al., 2008; Perrone et al., 2008). Previous studies indicated that stress-related transcription factors could translocate from the cytoplasm to the nucleus to induce the transcription of target genes in response to environmental stress, including Msn2p and Msn4p responses to various stress conditions (Görner et al., 1998; Roetzer et al., 2008), the Pap1p response to oxidative stress (Toone et al., 1998), and the Crz1p response to illumination (Bodvard et al., 2013). Similar to the above transcription factors, CgAsg1p-GFP migrated from the cytoplasm to the nucleus when the ambient pH decreased. In contrast to CgAsg1p, unexpectedly, CgHal9p-GFP was constitutively localized in the cytoplasm despite the change in ambient pH. These results indicated that the transcription factors CgAsg1p and CgHal9p use different mechanisms to promote acid-stress resistance, although further studies are needed to confirm this.

Subsequently, RNAseq was performed to investigate the transcriptional response to acid stress, and four leading ways were identified to be the main causes of sensitivity to acid stress in the *Cgasg1*Δ and *Cghal9*Δ strains. First, the mRNA abundances of many genes involved in the MAPK signaling pathway, including the pheromone signal transduction pathway and the HOG signaling pathway, were altered to varying degrees in the *Cgasg1*Δ and *Cghal9*Δ strains. The pheromone signal transduction pathway was weakened by the up-regulation of genes, including *GPA1*, which can exchange GDP to GTP, dissociate the β-γ dimer *STE4*-*STE18*, and prevent it from activating downstream effectors (Whiteway et al., 1989; Nomoto et al., 1990), as well as the final kinase coding gene *FUS3*, the phosphorylation of which could activate the mating pathway but suppress the filamentation pathway (Madhani et al., 1997). The HOG signaling pathway was also weakened by the down-regulation of the MAP kinase-encoding gene *HOG1* and a component of the regulatory system gene, *YPD1*, which control an array of adaptive responses (Kaloriti et al., 2012; Jandric et al., 2013). Second, the expression of genes implicated in plasma membrane or cell wall organization was reduced, thereby decreasing tolerance toward a low environmental pH (Kapteyn et al., 2001). Third, the transcription of several genes involved in trehalose accumulation were reduced, which is also an important cause of sensitivity to acid stress (Mahmud et al., 2010). Finally, the deletion of *CgASG1* or *CgHAL9* also lowered the transcription of *RIM101*, a key gene in the *C. glabrata* RIM101 signaling pathway, which was demonstrated to be required for maximal tolerance to weak acid-induced stress in *S. cerevisiae* (Mira et al., 2009). The RNAseq data were verified to be accurate by performing qRT-PCR on several genes involved in the aforementioned pathways. In conclusion, CgAsg1p and CgHal9p are required for tolerance of acid stress via the regulation of multiple pathways, such as the MAPK signaling pathway, plasma membrane or cell wall organization, trehalose accumulation, and the RIM101 signaling pathway.

To analyze the relationship between CgAsg1p and CgHal9p, qRT-PCR of *CgASG1*, and *CgHAL9* and localization of CgAsg1p-GFP and CgHal9p-GFP were performed, which indicated that the deletion of either gene did not influence the expression level and protein localization of the other. Additionally, the growth defect of the *Cgasg1*Δ strain in an acidic medium was reversed by the deletion of *CgHAL9*. Therefore, we deduce that *CgASG1* and *CgHAL9* function independently in acid-stress resistance, but the affects of the *CgASG1* and *CgHAL9* deletions could balance each other, as the deletion of both genes triggered other pathways that maintained pH homeostasis. Further studies are needed to confirm this.

Add it all up, the novel roles of transcription factors Asg1p and Hal9p played in acid-stress response were firstly studied in *C. glabrata* in this research. These results would lay a foundation for the establishment of the regulatory network under acid-stress conditions. Besides, the pyruvate production might be improved in the future studies by regulating the expression of *CgASG1, CgHAL9* or the target genes of Asg1p and Hal9p.

### Conflict of interest statement

The authors declare that the research was conducted in the absence of any commercial or financial relationships that could be construed as a potential conflict of interest.
