# Supplementary material for: Transcription factors Asg1p and Hal9p regulate pH homeostasis in Candida glabrata
Source: Front Microbiol. 2015 Aug 18;6:843. doi: 10.3389/fmicb.2015.00843 (PMC4539521; doi:10.3389/fmicb.2015.00843)
Supplement: Supplementary file 7 [file Presentation1.PDF]

Supplementary Material

Transcription factors Asg1p and Hal9p regulate pH homeostasis in *Candida glabrata*

Jing Wu[1, 2], Xiulai Chen[1, 2], Lijun Cai[1, 2], Lei Tang[2] and Liming Liu[1, 2 *]

[1] State Key Laboratory of Food Science and Technology, Jiangnan University, 1800 Lihu Road, Wuxi, Jiangsu, China
[2] The Key Laboratory of Industrial Biotechnology, Ministry of Education, Jiangnan University, 1800 Lihu Road, Wuxi, Jiangsu, China

*** Correspondence:** Liming Liu, State Key Laboratory of Food Science and Technology, Jiangnan University, 1800 Lihu Road, Wuxi, Jiangsu 214122, China.
e-mail: mingll@jiangnan.edu.cn

1. Supplementary Data

Supplementary Data 1. Up-regulated genes in the *Cgasg1*Δ and *Cghal9*Δ strains, compared with the wild-type strain in YNB medium.

Supplementary Data 2. Down-regulated genes in the *Cgasg1*Δ and *Cghal9*Δ strains, compared with the wild-type strain in YNB medium.

Supplementary Data 3. Up-regulated genes in the *Cgasg1*Δ and *Cghal9*Δ strains, compared with the wild-type strain in YNB-pH 2.0 medium.

Supplementary Data 4. Down-regulated genes in the *Cgasg1*Δ and *Cghal9*Δ strains, compared with the wild-type strain in YNB-pH 2.0 medium.

Supplementary Data 5. Up-regulated genes in *wt*, *Cgasg1*Δ and *Cghal9*Δ strains in YNB-pH 2.0 medium, compared with the corresponding strains in YNB medium.

Supplementary Data 6. Down-regulated genes in *wt*, *Cgasg1*Δ and *Cghal9*Δ strains in YNB-pH 2.0 medium, compared with the corresponding strains in YNB medium.

2. Supplementary Figures and Tables

2.1. Supplementary Tables

Table S1. The number of colonies in *C. glabrata* strains in YNB-pH 2.0 medium

	0 h	4 h	8 h	12 h
wt	$1.73 \pm 0.14 \times 10^7$	$4.13 \pm 0.30 \times 10^7$	$6.01 \pm 0.45 \times 10^7$	$3.69 \pm 0.48 \times 10^8$
Cgasg1Δ	$2.58 \pm 0.22 \times 10^7$	$1.00 \pm 0.08 \times 10^7$	$1.26 \pm 0.06 \times 10^6$	$6.45 \pm 0.61 \times 10^4$
Cgasg1Δ/CgASG1	$2.72 \pm 0.35 \times 10^7$	$6.58 \pm 0.42 \times 10^7$	$1.46 \pm 0.32 \times 10^8$	$7.83 \pm 0.25 \times 10^8$
Cghal9Δ	$5.11 \pm 0.17 \times 10^7$	$1.36 \pm 0.12 \times 10^7$	$2.04 \pm 0.26 \times 10^6$	$8.69 \pm 0.35 \times 10^4$
Cghal9Δ/CgHAL9	$3.02 \pm 0.26 \times 10^7$	$9.39 \pm 0.42 \times 10^7$	$3.85 \pm 0.67 \times 10^8$	$1.74 \pm 0.61 \times 10^9$

Table S2. Genes used in the validation of RNAseq data by qRT-PCR

Gene name	Protein	Fold change[a]	
		qRT-PCR	RNAseq analysis
CAGL0C04323g	Trehalase	0.70	0.16
CAGL0K03421g	Phosphoglucomutase 2	-1.56	-2.70
CAGL0I05940g	Phosphatidylinositol transfer protein SFH5	0.82	0.51
CAGL0I10384g	Polyamine transporter 2	0.43	0.11
CAGL0M13519g	ATP-dependent RNA helicase HAS1	0.21	1.34
CAGL0C03245g	Ubiquitin-like-conjugating enzyme ATG10	2.65	1.13
CAGL0I04180g	Metal-activated transcriptional activator protein	3.52	3.20
CAGL0B01243g	Mating-type-like protein ALPHA1	1.26	1.55
CAGL0G03597g	High osmolarity signaling protein SHO1	1.13	0.08
CAGL0B01232g	Ribosomal RNA small subunit methyltransferase NEP1	2.08	0.86
CAGL0E06534g	Nucleolar protein 9	2.27	1.43
CAGL0J05984g	Adenine deaminase	2.92	1.93

[a]Fold change: values represent fold change when *Cgasg1*Δ was treated with acid stress for 2 h.

2.2. Supplementary Figures

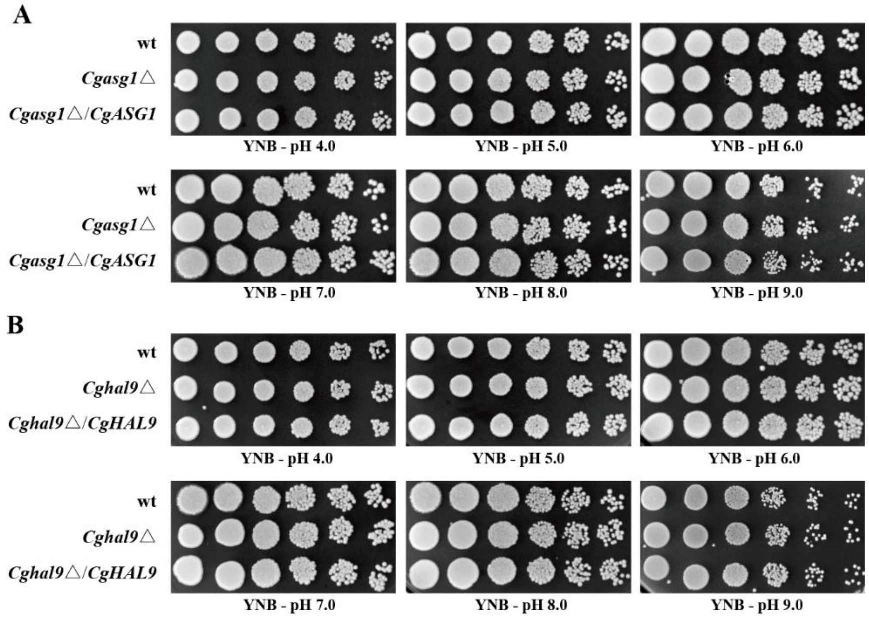

Figure S1. Growth assays on YNB media at pH 4.0-9.0. (A) Deletion of *CgASG1* has no effect on *C. glabrata* growth on YNB media at pH 4.0-9.0. (B) Deletion of *CgHAL9* has no effect on *C. glabrata* growth on YNB media at pH 4.0-9.0. Logarithmic-phase cells of each *C. glabrata* strain were adjusted to 2×10^7 cells/mL, and then 4 μL of serial tenfold dilutions were spotted onto the corresponding YNB media, as indicated. Pictures were taken after 4 days of growth at 30 ℃.

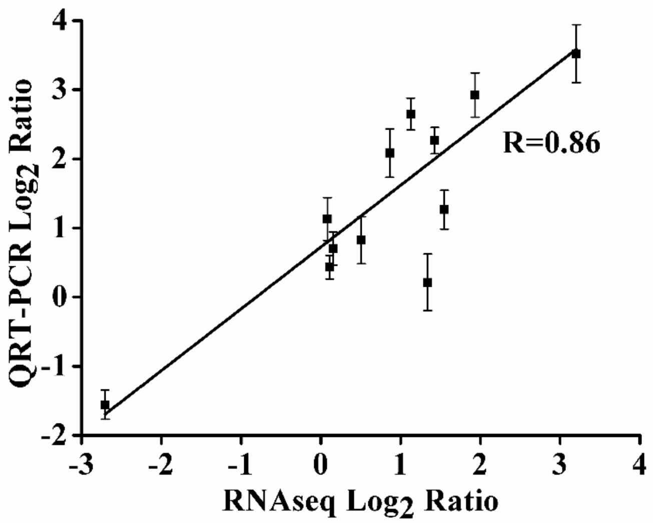

Figure S2. Validation of RNAseq data by qRT-PCR from YNB-pH 2.0 treated *Cgasg1*Δ strain.

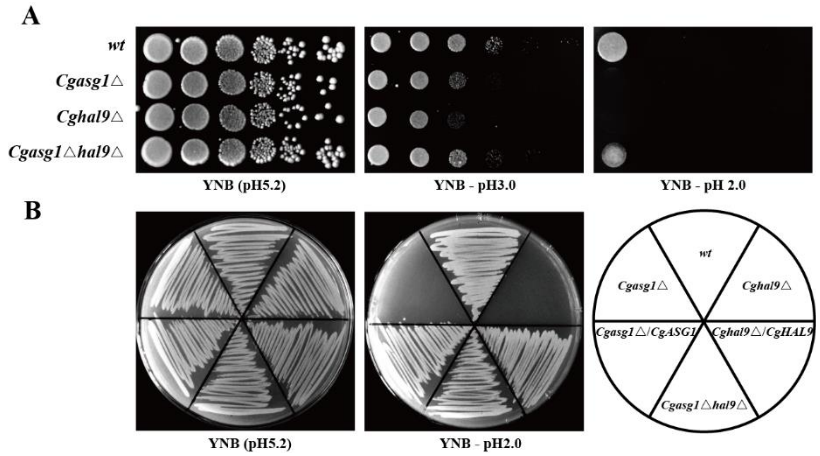

Figure S3. Effects of *CgHAL9* deletion on acid stress response in *Cgasg1*Δ background. (A) Logarithmic-phase cells of each *C. glabrata* strain were adjusted to 2×10^7 cells/mL, and then 4 μL of serial tenfold dilutions were spotted onto YNB plates at pH 2.0 and pH 3.0. Plates were incubated at 30 ℃ for 4 days. (B) Wild-type strain, mutant strains *Cgasg1*Δ and *Cghal9*Δ, reconstructed strains *Cgasg1*Δ/*CgASG1* and *Cghal9*Δ/*CgHAL9* and double mutant *Cgasg1*Δ*hal9*Δ were streaked on YNB and YNB-pH 2.0 plates. Pictures were taken after 4 days of growth at 30 ℃.
